# *HPRT1* Most Suitable Reference Gene for Accurate Normalization of mRNA Expression in Canine Dermal Tissues with Radiation Therapy

**DOI:** 10.3390/genes13111928

**Published:** 2022-10-23

**Authors:** Sang-Yun Lee, Yong-Ho Choe, Jang-Ho Han, Gunha Hwang, Moon-Yeong Choi, Gitika Thakur, Chan-Hee Jo, Seong-Ju Oh, Won-Jae Lee, Gyu-Jin Rho, Sung-Lim Lee, Tae-Sung Hwang

**Affiliations:** 1 College of Veterinary Medicine, Gyeongsang National University, Jinju 52828, Korea; 2 Yangsan S Animal Cancer Center, Yangsan 50638, Korea; 3 College of Veterinary Medicine, Kyungpook National University, Daegu 41566, Korea; 4 Research Institute of Life Sciences, Gyeongsang National University, Jinju 52828, Korea; 5 Institute of Animal Medicine, College of Veterinary Medicine, Gyeongsang National University, Jinju 52828, Korea

**Keywords:** radiation therapy, canines, reference genes, *HPRT1*

## Abstract

Reference genes are crucial in molecular biological studies as an internal control for gene re-search as they exhibit consistent expression patterns across many tissue types. In canines, radiation therapy is the most important therapeutic tool to cure various diseases like cancer. However, when using radiation for therapeutic strategy, radiation exposure to healthy tissues leads to some possible side effects such as acute radiation-induced skin injury and alters gene expression. Therefore, the analysis of a change in reference gene expression during the skin recovery process after radiation therapy is essential in healthy canine tissue. In the present study, we analyzed eight reference genes (*ACTB, GAPDH, YWHAZ, GUSB, HPRT1, RPL4, RPS5,* and *TBP*) in canine dermal tissues at 0, 1, 2, 3, 4, 5, 7, and 9 weeks of radiation exposure that affected the skin condition of canines. The stability of reference genes is determined by evaluating radiation therapy’s effect on healthy canine dermal tissue. Epidermal marker, *Keratin 10* expression varies each week after irradiation, and *HPRT1* is found to be the most suitable for normalization of mRNA expression in radiation-exposed canine dermal tissues. Changes in the gene expression level were evaluated by using a reliable tool such as quantitative real-time polymerase chain reaction (qRT-PCR). In order to achieve a valid qRT-PCR result, the most stable reference genes used for normalization after the radiation exposure process are important. Therefore, the current study was designed to evaluate the most stable reference gene for the post-irradiation canine tissues. After radiation exposure, the alternation of reference gene expression was estimated by three algorithms (geNorm, Normfinder, and Bestkeeper). The RG validation programs (GeNorm and NormFinder) suggested that *HPRT1*, *RPL4*, and *TBP* were suitable for normalization in qRT-PCR. Furthermore, three algorithms suggested that *HPRT1* was the most stable reference gene for normalization with qRT-PCR results, regardless of before and after radiation exposure. Whereas *GAPDH* was found to be the most unstable reference gene. In addition, the use of stable or unstable reference genes for the normalization of *Keratin 10* expression showed statistical differences. Therefore, we observed that, to obtain accurate and suitable PCR results of the canine tissues with and without radiation exposure, the *HPRT1* reference gene is recommended for normalization with its high stability. Additionally, the use of RGs such as *HPRT1, RPL4*, and *TBP* for normalization in qRT-PCR experiments is recommended for post-radiation canine tissues to generate more accurate and reliable data. These results will provide fundamental information regarding internal controls for gene expression studies and can be used for the analysis of gene patterns in regenerative medicine.

## 1. Introduction

Radiation therapy is commonly used for the treatment of cancer in veterinary medicine [1]. In dogs, radiation therapy is a reliable treatment for various types of cancers, such as vertebral osteosarcoma, non-lymphomatous nasal tumor, and intracranial meningiomas [2,3,4]. However, radiation therapy-induced skin changes are serious side effects of radiation therapy, such as radiation dermatitis [5]. Also, another side effect in molecular biology occurs in radiation-induced skin injury, such as alternation in oxidative stress and arrest in cell cycle [6,7,8]. Irradiation also causes DNA damage and changes the gene expression in the cells [9]. Hence, due to the side effect of radiation therapy on cellular and gene expression, evaluating the change in gene expression in radiated skin tissue is essential for understanding the complexity of post-irradiation reactions.

The qRT-PCR is the most reliable and widely used tool for the quantification of a specific mRNA [10]. It is easy to use for obtaining results and reliable data due to no post-PCR handling. To compare the expression of the gene of interest (GOI) in many experiments using qRT-PCR, the method of normalization using a suitable reference gene is essential. Reference genes play an important role in the maintenance of cell functions. The traditional reference genes such as glyceraldehyde-3-phosphate dehydrogenase gene (*GAPDH*), β−actin gene (*ACTB*), and TATA−binding protein gene (*TBP*) are generally used reference genes for analysis of gene expression [11,12,13]. However, it has been reported that the stability of the reference gene is variable in experimental conditions, and there is no universal reference gene that can be applied to all conditions [14,15]. Therefore, the selection of the most stable reference gene is an essential step in various conditions, such as radiation exposure. The suitable reference genes in canine tissue have been studied in previous studies [16,17]. The genetic analysis of radiation-induced gene expression changes and the effect of irradiation on the stability of reference genes in human were investigated [18,19]. However, no previous studies have been conducted to estimate the stability of reference genes in the treatment of canine radiotherapy.

DNA damage caused by radiotherapy can be due to the direct or indirect action of radiation [20]. In the direct action, the molecular structure of DNA was directly damaged by the radiation. The change of molecular structure prompts cell damage. In the indirect action, the radiation generates free radicals such as hydroxyl and alkoxy derived from water and cellular molecule in the cell. The free radical reaction with DNA molecules causes molecular structural damage and impairment of cellular function. DNA damage caused by radiation therapy affected the change of gene expression patterns, including reference genes. However, no reference gene has been known to be perfectly stable in all conditions. Especially, the normalization of qRT-PCR results using unstable and inadequate reference genes was shown unreliable or contradictory results [21,22]. Therefore, validation of stable reference genes under experimental conditions is necessary to obtain reliable qRT-PCR results [23]. Therefore, the aim of this study was to estimate the most stable reference gene from the post-irradiation sample for normalization of the qRT-PCR results.

After radiation exposure to healthy canine skin, we assessed the stability of eight reference genes with three algorithms (geNorm, Normfinder, Bestkeeper). In geNorm, the average pair-wise variation of candidate reference gene expression is compared to all other evaluated reference genes. The Normfinder was compared in all sample’s cycle threshold values (Ct values) via analysis of variance and was utilized for evaluating expression value. The Bestkeeper produced Bestkeeper index combined the geometric mean of Ct values in all samples reference gene. The Bestkeeper index was compared in a pair-wise fashion via Pearson correlation coefficients for evaluating the reference gene stability ranking [24]. Previously, we analyzed the most stable reference gene in other species using three algorithms [21,22,25]. Eight candidate reference genes were estimated by independent statistical algorithms, different stability ranking was obtained. As a result of all collected algorithms, this study suggested a stable expression of reference genes to obtain reliable conclusions regarding post-irradiation changes in gene expression.

## 2. Materials and Methods

### 2.1. Ethics Approval and Animals

All procedures were approved by the research ethics committee of Gyeongsang National University Animal Center for Biomedical Experimentation (GNU-200916-D0061). This study was performed using a total of four male beagles. Beagle had a weight of 8.7 kg and an age of 55 weeks old at time of irradiation.

### 2.2. Expose Irradiation and Collect Sample

The dogs have fasted for 12 h before anesthesia. Anesthesia was induced with propofol (6 mg/kg, intravenous; Myungmoon pharm, Seoul, Korea) administration. General anesthesia was maintained with isoflurane (Hana Pharm, Kyonggido, Korea) and oxygen (2 L/min) via endotracheal intubation. All dogs were positioned in right lateral recumbency and irradiated to the left flank area. The irradiated skin was covered with a standard commercial thickness bolus of 1 cm to improve the dose distribution for treatment. Irradiation was performed with megavoltage radiation delivered with a linear accelerator (Elekta Synergy, Elekta AB, Stockholm, Sweden) using a 6 MeV electron. The dogs received 12 fractions of 4 Gy for a total dose of 48 Gy daily for 12 days with an area of 14 cm × 14 cm. The skin tissue was collected by twice punch biopsy at 0, 1, 2, 3, 4, 5, 7, and 9 weeks after radiation. Control skin tissue was collected from the opposite side of the irradiated flank area. The skin sample was collected in 1.5 mL Eppendorf tubes for isolating RNA.

### 2.3. Candidate Reference Gene and Primer

Based on various intracellular functional activities, eight reference genes were selected. All reference gene primers were designed by Primer 3 Plus software and confirmed not to make homodimers, heterodimers, and hairpins by OligoAnalayzer 3.1 software. Primers’ full name, sequence, and amplicon size are listed in Table 1. To validate the PCR efficiencies, a standard curve of each primer was generated from the Ct values used by a four-fold serial dilution of normal tissue-derived cDNA. The values related to correlation (R^2^), standard curve slope (M), intercept (B), and PCR efficiencies (E) were estimated using RoterGene Q Series Software (Qiagen, Hilden, Germany).

### 2.4. RNA Isolation, cDNA Synthesis, and qRT-PCR

After all sample collection, RNA was extracted by RNeasy Mini Kit (Qiagen, Hilden, Germany). In order to eliminate DNA contamination, RNase-free DNase treatment step was performed. To select uncontaminated RNA, RNA quality at A260/280 ratio were evaluated by OPTIZEN NanoQ Lite spectrophotometer. To synthesize cDNA, 1 μg of RNA sample was used by Omniscript Reverse Transcription Kit (Qiagen, Germany) at 37 °C for 1 h. PCR amplification was achieved by using Rotor-gene (Qiagen, Germany). qRT-PCR was performed by using Rotor-Gene Q with Rotor gene with a reaction mix containing 2X SYBR Green mix (Qiagen, Germany), 2 μL cDNA, 0.7 μM forward and reverse primers.

The qRT-PCR follows the program of pre-denaturation, denaturation, and combined annealing/extension step. Each step was comprised of a different temperature and setting time. Pre-denaturation at 95 °C for 2 min; 45 PCR cycles at 95 °C for 10 s, 60 °C for 6 s. The melting curve shows temperature alters from 60 °C to 95 °C by 1 °C per s. After that, the temperature was cooled down at 40 °C for 30 s according to the qRT-PCR program with a minor modification in the manufacturer’s protocol. Melting curves and Ct values were analyzed using Rotor-Gene Q Series Software (Qiagen, Germany). Further, gel electrophoresis was used to confirm the expected product size of each product from qRT-PCR and to check out nonspecific amplification. A 1% agarose gel was used, and images were analyzed using zoom browser EX5.7 software (Canon, Japan).

### 2.5. Determination of Stable Reference Gene Expression

Ct values of the reference gene for each skin sample were analyzed by three kinds of algorithms (geNorm, Normfinder, and Bestkeeper). The geNorm assesses the stability of expression (M value) between each gene. The reference gene possessed the largest M value that meaning the least stable reference gene and is removed from the calculating pool, the new pool is recalculated for a new M value. Finally, only two reference genes with the minimum M value were evaluated. In addition, geNorm determines the optimal number of a reference gene for the normalization factor (normalization factor, NF) by continuously estimating the pairwise variation (V_n/n+1_) between consecutively ranked NF (NF_n_ and NF_n+1_) [15]. To check the correlation between the optimal number of reference genes (NF_opt_) and the three most stable reference genes (NF_3_), Pearson’s correlations of normalization factors were analyzed by SPSS to reduce the excessive number of reference genes during normalization. The Microsoft-Office Compatible Excel Macro tool for using the geNorm algorithm is available at http://ulozto.net/xsFueHSA/genorm-v3-zip (accessed on 8 August 2022) [24]. Normfinder is based on a Visual Basic application (VBA) program to calculate intra and inter-group variation to suggest the lowest value reference gene means the most stable reference gene. In addition, Normfinder presents the best combination of two genes for normalization [26]. The Normfinder plug-in is available at http://moma.dk/normfinder-software [24]. The Bestkeeper, based on Microsoft Excel, evaluates comparative analysis based on Pearson’s pairwise correlations of all reference genes against each other and calculates the standard deviation (SD) [15]. The Bestkeeper result, with an SD > 1.0 was regarded as an unusable reference gene and a lower SD value was considered a stable reference gene [27]. Bestkeeper applications, data processing, and required information are available to download at http://www.gene-quantification.de/bestkeeper.html [24].

### 2.6. The Use of Different Reference Gene in the Normalization of Gene of Interest

Various reference genes, including the most stable RGs in the current investigation, were used to assess the impact of reference gene stability on the normalization of GOI, and the traditional reference genes which are demonstrated as a less stable reference gene for calculating relative gene expression of *Keratin 10* (Table 1). *Keratin 10* gene expression was evaluated by the qRT-PCR as described above. Expression of *Keratin 10* was normalized to the most stable reference gene and the least stable reference gene using RotorGene Q Series Software (Qiagen, Germany).

### 2.7. Statistical Analysis

Data analysis was performed using PASW Statistics 18 (SPSS Inc., Chicago, IL, USA). Statistical significance was determined by a one-way (ANOVA) analysis of variance followed BY the Bonferroni post hoc test. All data were reported as mean ± SD, and differences were regarded as significant when *p* < 0.05.

## 3. Results

### 3.1. Exposure to Radiation in Healthy Canine Skin

**Figure 1:**
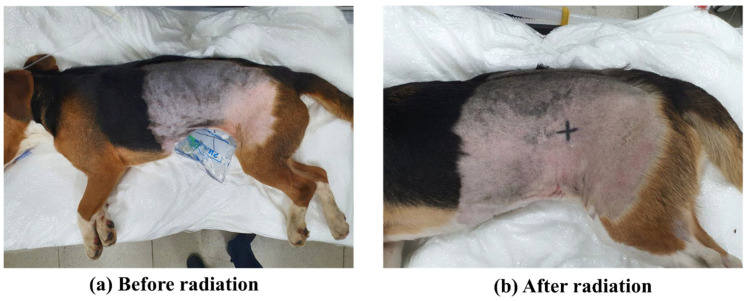
The healthy beagle received 12 fractions of 4 Gy for a total dose of 48 Gy daily for 12 days. After irradiation, the beagle’s skin was observed immediately. With the exception of slight erythema, the skin showed no significant difference.

The healthy dogs were given a total dosage of 48 Gy in 12 portions of 4 Gy per day for 12 days at the left trunk skin. After radiation therapy, the dog’s skin was observed immediately (Figure 1). During radiation therapy for 12 days, there were no notable changes in the skin tissue stimulated by radiation except faint erythema.

### 3.2. Evaluation of Amplicon Size, Ct Values, and Primer Efficiency of Selected Reference Genes

**Figure 2:**
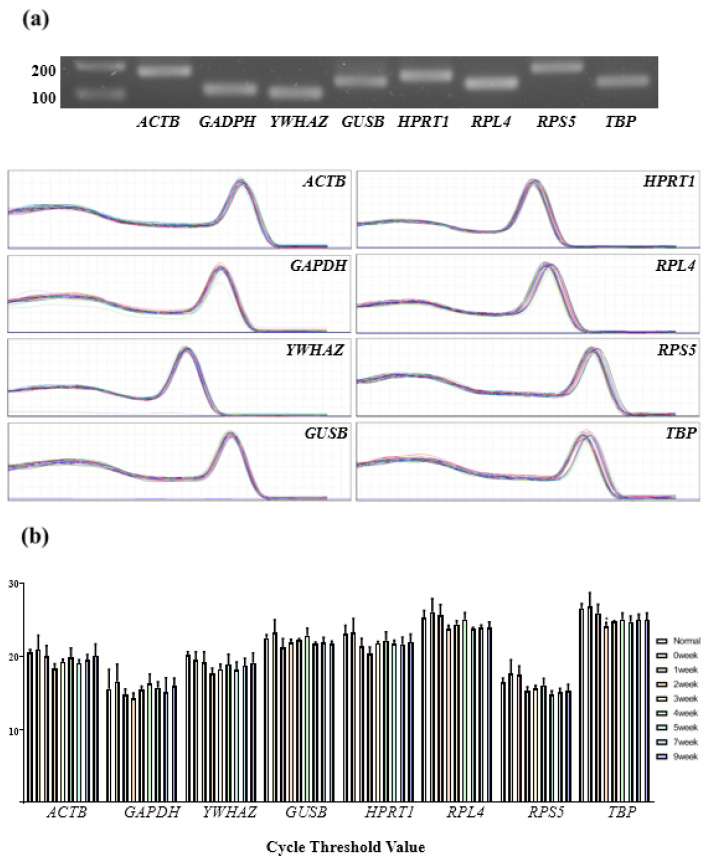
Examination of amplicon size, melting curve, and Ct value of candidate reference gene. (**a**) PCR amplicon product was evaluated by electrophoresis using 1% agarose gel. Melting curves and agarose gel electrophoresis were used to examine the specificity of primers and amplicon lengths of eight chosen reference genes. Electrophoresis used 1% agarose gel shown the expected product size without non-specific amplification. Lanes show ladder (100 and 200 bp). During melting curve analysis, a single peak of melting curve informed with no non-specific product. (**b**) The Ct value of eight reference genes in 0, 1, 2, 3, 4, 5, 7, and 9 weeks post-irradiation. The data was presented as mean ± SD. *p* value by one-way (ANOVA) analysis of variance followed BY Bonferroni post hoc test were * *p* < 0.05.

For the analysis of the melting curve in this study, each reference gene amplification was demonstrated by a single product without any hairpin and dimers amplification due to a single peak melting curve. In addition, the gel electrophoresis of amplicons was shown an expected product size without nonspecific amplification, such as hairpin, self-dimer, and heterodimer (Figure 2a). In addition, the primer efficiency was tested using a four-fold serial dilution of cDNA, which produced 0.97–1.03 PCR efficiencies (E) and a 0.991–0.997 correlation coefficient (R^2^), implying that the eight candidate reference gene primers were suitable for qRT-PCR (Table 1). To estimate the mRNA transcription level for each sample, all reference gene Ct values were evaluated, as shown in Figure 2b. After exposure to radiation, each week’s sample showed a different Ct value. The Ct value of *TBP* at 2 weeks was significantly (*p* < 0.05) different.

### 3.3. Analysis of the Most Stable Reference Gene by geNorm

**Figure 3:**
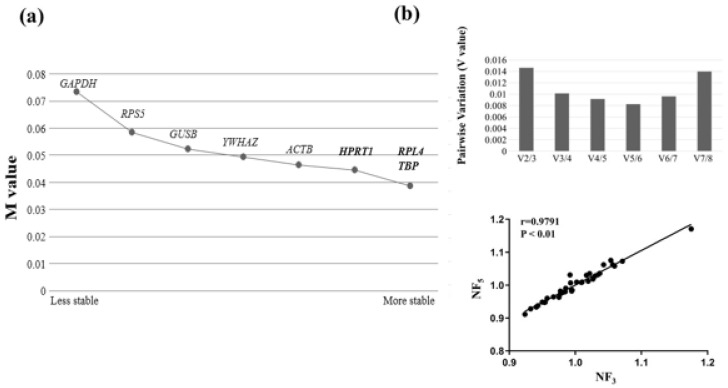
Assessment for the stability of reference gene used geNorm. (**a**) The graph presents the ranking of stability of the reference gene. The right side of the graph represents the most stable reference gene, and the left side of the graph represents the most unstable reference gene. The best of three stable reference genes was shown in bold letters. (**b**) The graph shows the optimal number of reference gene at normalization (NFopt). NFopt was present 5 reference gene (NF5) by pairwise variation (V5/6). Between NF3 and NF5, Pearson’s correlations of normalization factor analyzed by SPSS was suggest high correlation (r = 0.9791, *p* < 0.01).

GeNorm was used to examine the Ct values of radiation-exposed canine dermal tissues and provide an M value and stable ranking of 8 reference genes through progressive stepwise removal of the least stable reference genes. All reference genes had an M value of less than 1.5, indicating that all genes were reliable to use as reference genes. The geNorm results suggested that *RPL4* and *TBP* were the most stable reference genes and the second one was *HPRT1, GAPDH* and *ACTB*, which were generally used as traditional genes, appeared to be less stable genes (Figure 3a). Particularly, *GAPDH* was estimated as the most unstable reference gene. Moreover, the pairwise variation by geNorm showed that the optimal number of reference genes proposed five reference genes for normalization (NF_5_). The correlation of the normalization factor between five reference genes, which is the optimal number (NF_5_), and the most stable reference gene (NF_3_) was analyzed to remove the excessive usage of the reference gene. A high correlation (r = 0.9791, Pearson) was observed between NF_5_ and NF_3,_ indicating that the three most stable reference genes were sufficient for normalization for qRT-PCR.

### 3.4. Analysis of the Most Stable Reference Gene by Normfinder

**Figure 4:**
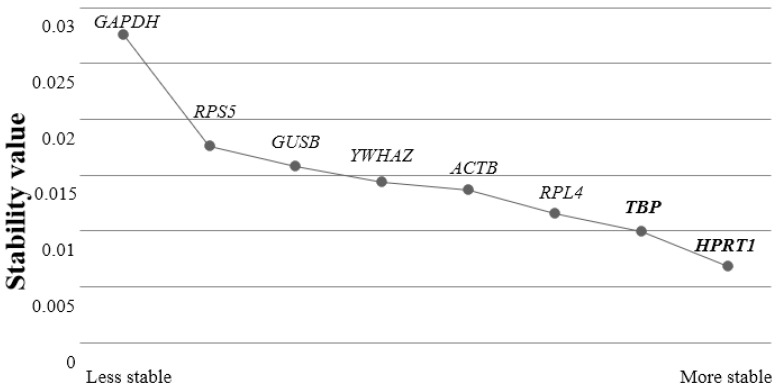
Analysis of reference gene stability used Normfinder. The most stable reference gene ranked on the right side of the graph and the most unstable reference gene ranked on the left side of the graph. The best combination of two reference genes was shown in bold letters.

After all reference genes were analyzed by Normfinder, *HPRT1* and *TBP* were determined as the most stable reference gene. Moreover, the Normfinder algorithm showed the best combination of the two most stable reference genes for normalization *HPRT1* and *TBP* (Figure 4). This analysis showed similar results as geNorm algorithm where *HPRT1* and *TBP* were more stable reference genes while generally used traditional reference genes *ACTB* and *GAPDH* expression were unstable. Additionally, results demonstrated that the *GPADH* was the most unstable reference gene as analyzed by the geNorm software.

### 3.5. Analysis of the Most Stable Reference Gene by Bestkeeper

Bestkeeper was used to further investigate the eight reference genes expression stability, and the R-value was determined to measure the stability. The most stable reference gene calculated by Bestkeeper presents the lowest CV (CV ± SD), where SD higher than 1 should not be considered [28]. The SD Value of all the reference genes except *GAPDH* was less than 1. *GAPDH* with an SD value greater than 1 was suggested as an unacceptable reference gene for normalization. In addition, *GUSB* and *HPRT1* were the most stable reference gene with the lowest SD ± Ct values (Table 2). Moreover, the SD ± Ct values of *GUSB* were less than that of *HPRT1*, makes *GUSB* a more stable reference gene. A small difference in stable rankings of 8 reference genes for geNorm, NormFinder, and Bestkeeper may be due to using the different algorithms.

### 3.6. Use of Most Stable Reference Genes for Normalization

**Figure 5:**
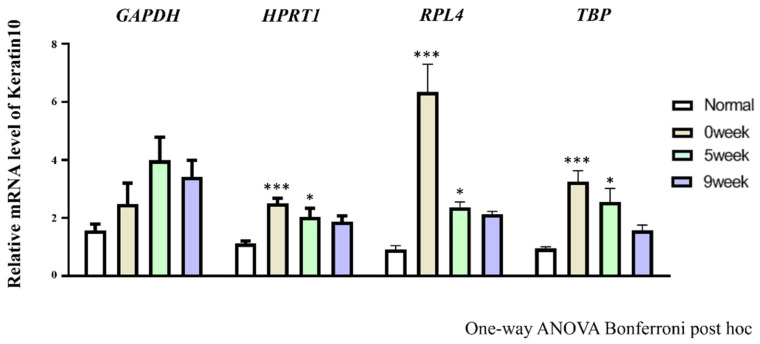
The application to normalization of estimated reference gene. The normalization used stable (*HPRT1, RPL4* and *TBP*) or the least stable (*GAPDH*) reference gene. *Keratin 10* gene was normalized by *HPRT1, RPL4, TBP* and *GAPDH* to prove the influence of reference gene stability. Significant (* *p* < 0.05; *** *p* < 0.0001) differences in *Keratin 10* expression level between normal and post irradiation skin are presented as an asterisk.

In our results, the most stable reference gene *HPRT1*, and the most unstable reference gene *GAPDH* were suggested by the analysis of three algorithms (Table 3). Reference gene was used for the normalization of *Keratin 10* gene, a common epidermal marker in each group of the post-irradiation canine tissues (Figure 5). When *HPRT1, RPL4, TBP* and *GAPDH* were used for normalization, the expression of *Keratin 10* was changed in each sample after radiation exposure every week (Figure 5). The stable reference gene used for normalization of *Keratin 10* expression showed significantly upregulated expression at 0 week and 5th week compared to the normal tissue. However, in the case of *GAPDH*, no significant difference in *Keratin 10* expression was observed in all the samples every week after irradiation. In addition, *Keratin 10* expression was highly increased at 0 week using *HPRT1.*

## 4. Discussion

As radiation therapy is currently used for the treatment of cancer and qRT-PCR was the usable tool for estimating the radiation therapy reaction on biomarkers [29]. The use of stable reference genes in qRT-PCR is an essential factor that informed the change in gene expression. Previous studies in humans have been conducted to evaluate the most stable reference gene and change of gene expression under the irradiation condition [18]. Alternation of gene expression during recovery of radiation-induced side effects must be studied. Hence, the aim of this study was to evaluate the eight-reference gene stability in canine post-irradiation conditioned skin tissue through widely used three algorithms (geNorm, Normfinder, and Bestkeeper).

During the evaluation of the reference gene stability, the factors such as the size of the primer and amplicon can have an impact on the reference gene stability [16]. Therefore, in the present study, primers with amplicon size between 100 and 200 were used to avoid additional effects on the reference gene stability. As a result, geNorm and Normfinder are most commonly used in several reports for reference gene analysis studies [30,31]. In this study, the analysis results of geNorm and Normfinder were almost similar, and these two algorithms suggested *TBP, HPRT1* and *RPL4* as the most stable reference genes. On the other hand, *HPRT1* was found to be the most stable reference gene considering all three algorithms; the different ranking of reference genes in Bestkeeper is possibly generated by a different algorithm than other two algorithms [31,32].

Under normal skin conditions, epidermal stem cells routinely divide to replace dead skin tissue; however, this process was inhibited by irradiation, resulting in normal skin epithelial cell death [33]. Since the epidermis is radiosensitive tissue, determining side effects at the molecular level is critical for observing clinical skin changes and the biological state of the injured epidermis. In our study, 4 Gy radiation exposure was given daily to healthy skin for 12 days. Faint erythema was observed in all beagles during 12 days of irradiation. These skin symptoms are already well known as the most common side effects of radiation therapy. In addition, healthy skin after radiation exposure follows some possible side effects, such as erythema, desquamation, pigmentation, and ulceration [28]. Although the side effect of radiation exposure is well known in clinical studies, the analysis of change in gene expression of long-term post-irradiation in canine skin is poorly understood. This study was designed to estimate the alternation of gene levels in canine skin after radiation therapy.

In molecular biology, qRT-PCR was the most reliable and widely used tool for the quantification of a specific mRNA [10]. Previously, studies have been conducted to analyze the expression of canine gene levels using PCR after radiotherapy [26,29]. However, alteration of reference gene expression during recovery from radiation therapy is not clear. To test the influence of irradiation on the reference gene expression, each skin tissue was analyzed at 0, 1, 2, 3, 4, 5, 7, and 9 weeks post-irradiation. The result in Figure 2 showed a change in the Ct value. Ct value was calculated by using the basic equation [XT = X0 × (1 + E) Ct], where XT is the number of molecules at the threshold cycle, X0 is the number of molecules, and E is the efficiency of amplification] [14]. It is observed that the Ct value of eight reference genes was not constant due to the radiation exposure. Especially, alternation in gene expression was a complex response to deal with post-irradiation damage [19]. In this study, the variation in Ct value is consistent with the results of previous studies, where radiation affects the expression of genes [15]. Therefore, irradiation affected the expression of the reference gene, which leads to a change in the Ct value.

The Ct value for eight reference genes was estimated for the stability of reference genes using the most well-known three algorithms from samples at 0, 1, 2, 3, 4, 5, 7, and 9 weeks post-irradiation. Consequently, *HPRT1* was evaluated as the most stable reference gene through three algorithms, where *GAPDH* and *ACTB,* which is a traditional reference gene, were observed as the less stable. The enzyme encoded hypoxanthine phosphoribo-syltransferase-1, *HPRT1*, catalyzing the conversion of hypoxanthine and guanine to their respective mononucleotides induced pruin salvage pathways [27]. Consistent with this result for ranking a reference gene, *HPRT1* was observed as the stable gene in canine sample analysis with various experimental conditions, including healthy and dystrophic muscle [24] and myeloid-derived suppressor cells [34]. *GAPDH* and *ACTB* which are traditional reference genes and most popularly used in normalization. However, *GAPDH* was found to be unstable in the present study, which is consistent with the result of the previous studies [35,36]. In addition, it was reported that the traditional reference gene was unstable under the post-irradiation condition [37]. On the other hand, other studies with other sample analysis such as canine hindbrain [38] and canine oral tumor [39] estimated that *GAPDH* and *ACTB* were stable reference genes, respectively. It suggested that the discrepancy in the ranking of the reference gene was caused by differences in the experimental condition. Hence, before analyzing gene expression by qRT-PCR, selection of the most stable reference gene under particular experimental conditions is an essential step [22,25].

In the present study, we evaluated the effect of radiation on the stability of reference genes in canines during normalization and emphasized the possibility of controversial results using traditional reference genes without validation. The application to normalize the *Keratin 10* was analyzed by three algorithms, the most stable gene, *HPRT1*, and the most unstable gene, *GAPDH*. *Keratin 10* is an important structural protein that controls dermal cell growth, differentiation, and migration during wound healing and tissue regeneration [40,41,42]. In previous studies, it is well known that *Keratin 10* expression was changed by radiation exposure [43,44]. Although the result presents the same tendency, normalization with *HPRT1* observed a significant difference but normalization with *GAPDH* showed no significant difference, in statistical analysis. Previously, the increase in *Keratin 10* due to radiation exposure has been reported in mice and humans [45,46]. Therefore, from the results of this study, we suggest that *HPRT1* is the most stable reference gene in radiation-irradiated skin tissue. During quantitative real-time PCR analysis, 2 and 3 (*RPL4, TBP*) ranked RGs presented in this study will together produce more accurate results than using *HPRT1* alone as a reference gene. These findings suggest the importance of validation of reference genes before normalization. Without validation of reference gene stability, the use of traditional reference genes can lead to inaccurate or controversial results [21,22].

## 5. Conclusions

In conclusion, the present study demonstrated that the three RGs, *HPRT1, RPL4*, and *TBP* among the pool of eight RGs are suitable for normalizing the expression of GOI and that the traditional RG such as *GAPDH* is identified as less stable under current experimental conditions. Also suggested that *HPRT1* was evaluated as the most stable reference gene for gene expression study with a post-irradiation on canine skin reaction. In particular, it is revealed that *GAPDH*, which is commonly used for the previous related study, exhibits the most unstable expression out of eight reference gene pool. In order to derive more reliable results for the related studies to be carried out in the future, our results also provide a foundation for the further use of RT-qPCR in the analysis of gene expression in radiation therapy for canines.

## Figures and Tables

**Table 1:** Lists of candidate reference genes and their information.

	Information of Primers		Standard Curve Parameters				
Gene Name (Symbol)	Sequence	Base Pair	Accession	R^2^	M	B	E
β−actin (*ACTB*)	F : GCACTCTTCCAACCTTCTTTCC	179	AF021873.2	0.993	−3.489	34.252	1.02
	R : GCTGTGATTTCCTTCTGCATCC						
Glyceraldehyde-3−phosphate dehydrogenase (*GAPDH*)	F : GGAGAAAGCTGCCAAATATGACG	118	NM_001003142.2	0.991	−3.436	35.514	0.98
	R : ACTGTTGAAGTCACAGGAGACC						
Tyrosine 3−monooxygenase/tryptophan 5−monooxygenase activation protein, zeta polypeptide (*YWHAZ*)	F : GTGAAGAGTCATACAAAGACAGCA	110	XM_014118550.1	0.992	−3.511	37.145	1.01
	R : CCCTCCTTCTCCTGCTTCAG						
β−glucuronidase (*GUSB*)	F : ATCTGTAGTCATGTGGTCTGTAGC	149	AF019759.1	0.996	−3.332	33.257	0.99
	R : GGTCTGCTTCATAGTTGGAATTGG						
Hypoxanthine phosphoribosyl transferase 1 (*HPRT1*)	F : GACTGAAGAGCTACTGTAATGACC	168	NM_001003357.2	0.996	−3.412	36.915	0.98
	R : TCTTTGGATTATGCTCCTTGACC						
Ribosomal protein 4 (*RPL4*)	F : AATGAGAAACCGTCGTCGTATCC	141	NM_001252409.1	0.992	−3.355	39.041	1.01
	R : GGAGCAAGTTTCAGAATGTTCAGC						
Ribosomal protein S5 (*RPS5*)	F : TGAAGGAGAAGTATGCCAAGTACC	188	XM_533568.5	0.995	−3.435	39.145	0.97
	R : GAGCAGATGGATGATCTCGAAGG						
TATA box binding protein (*TBP*)	F : ATCTGGTATCCCTTACGCTTCG	137	XM_849432.4	0.995	−3.498	36.972	1.03
	R : GCAAGAGAGTCTGGTTTGTTTCC						
*Keratin 10*	F : CTCGTGACTACAGCAAATACTACC	105	NM_001013425.1	0.997	−3.501	33.783	1.01
	R : TGGCATTGTCGATCTGAAGC						

**Table 2:** Stability of reference gene analyzed by Bestkeeper.

	*GAPDH*	*YWHAZ*	*TBP*	*RPS5*	*RPL4*	*ACTB*	*HPRT1*	*GUSB*
SD [±CT]	**1.198596**	0.993889	0.95946	0.950324	0.93821	0.935185	0.901281	0.654506
CV [% CT]	**7.732737**	5.277597	3.791541	5.959286	3.811451	4.742051	4.112048	2.958517

Identification of expression of reference gene stability used Bestkeepr. The most stable reference gene to be placed the right side of table and the most unstable reference gene to be placed the left side. The data present coefficient of variance (CV s.e.m.) and SD (±Ct) values. The reference gene of presenting a s.e.m higher than 1 was shown in bold letter.

**Table 3:** Rank of reference gene stability all algorithms.

	*ACTB*	*GAPDH*	*YWHAZ*	*GUSB*	*HPRT1*	*RPL4*	*RPS5*	*TBP*
geNorm	4	8	5	6	3	1	7	1
Normfinder	4	8	5	6	1	3	7	2
Bestkeeper	3	8	7	1	2	4	5	6
Total Rank	4	8	6	5	1	2	7	3

## Data Availability

Not applicable.

## Abbreviations

ACTB	β-actin
CV	Coefficient variance
GAPDH	Glyceraldehyde-3-phosphate dehydrogenase
GOI	Gene of interest
GUSB	β-glucuronidase
HPRT1	Hypoxanthine phosphoribosyl transferase 1
qRT-PCR	Quantitative real-time polymerase chain reaction
RPL4	Ribosomal protein 4
RPS5	Ribosomal protein S5
SD	Standard deviation
TBP	TATA box binding protein
VBA	Visual Basic application
YWHAZ	Tyrosine 3-monooxygenase/tryptophan 5-monooxygenase activation protein, zeta polypeptide
